# Role of computed tomography in the evaluation of regional metastasis in well-differentiated thyroid cancer

**DOI:** 10.3389/fradi.2023.1243000

**Published:** 2023-10-31

**Authors:** Richa Vaish, Abhishek Mahajan, Nilesh Sable, Rohit Dusane, Anuja Deshmukh, Munita Bal, Anil K. D’cruz

**Affiliations:** ^1^ Head and Neck Services, Tata Memorial Hospital, Homi Bhabha National Institute, Mumbai, India; ^2^ Department of Radiodiagnosis, Tata Memorial Hospital, Homi Bhabha National Institute, Mumbai, India; ^3^ Department of Statistics, Tata Memorial Hospital, Homi Bhabha National Institute, Mumbai, India; ^4^ Department of Pathology, Tata Memorial Hospital, Homi Bhabha National Institute, Mumbai, India

**Keywords:** thyroid cancer, lymph node metastasis, lymphatic metastasis/diagnostic imaging, ultrasonography, computed tomography

## Abstract

**Background:**

Accurate neck staging is essential for performing appropriate surgery and avoiding undue morbidity in thyroid cancer. The modality of choice for evaluation is ultrasonography (US), which has limitations, particularly in the central compartment, that can be overcome by adding a computed tomography (CT).

**Methods:**

A total of 314 nodal levels were analyzed in 43 patients with CT, and US; evaluations were done between January 2013 and November 2015. The images were reviewed by two radiologists independently who were blinded to histopathological outcomes. The sensitivity, specificity, negative predictive value (NPV), positive predictive value (PPV), and accuracy of US, CT, and US + CT were calculated using histology as the gold standard.

**Results:**

The overall sensitivity, specificity, PPV, and NPV for US, CT, and US + CT were 53.9%, 88.8%, 74.1%, and 76.4%; 81.2%, 68.0%, 60.1%, and 85.9%; and 84.6%, 66.0%, 59.6%, and 87.8%, respectively. The overall accuracy of the US was 75.80%, the CT scan was 72.93%, and the US + CT scan was 72.93%. For the lateral compartment, the sensitivity, specificity, PPV, and NPV for the US, CT, and US + CT were 56.6%, 91.4%, 77.1%, and 80.5%; 80.7%, 70.6%, 58.3%, and 87.8%; and 84.3%, 68.7%, 57.9%, and 89.6%, respectively. The accuracy of the US was 79.67%, the CT scan was 73.98%, and the US + CT scan was 73.98% for the lateral compartment. For the central compartment, the sensitivity, specificity, PPV, and NPV for the US, CT, and US + CT were 47.1%, 76.5%, 66.7%, and 59.1%; 82.4%, 55.9%, 65.1%, and 76.0%; and 85.3%, 52.9%, 64.4%, and 78.3%, respectively. The accuracy of the US was 61.76%, the CT scan was 69.12%, and the US + CT scan was 69.12% for the central compartment.

**Conclusions:**

This study demonstrated that CT has higher sensitivity in detecting nodal metastasis; however, its role is complementary to US due to low specificity.

## Introduction

Well-differentiated thyroid cancers (WDTC) have high survival rates, and avoiding morbidity is of utmost importance in these patients. Thyroid surgery is intricate, and the outcomes vary according to the experience of the surgeons and the volume of the surgical center (1). Appropriate surgical management is essential for achieving a high cure rate and avoiding unnecessary morbidity. The extent of both central and lateral neck dissection is a matter of debate. Although global guidelines recommend therapeutic neck dissection for patients with confirmed regional metastasis (2–4), there is still no consensus on prophylactic neck dissection (Table 1) (5–12). Table 1 presents a summary of the meta-analyses on central compartment lymph node dissection. The regional recurrence is one of the most important factors that warrant thyroid carcinoma re-surgery. The incidence of regional recurrence has been reported to range from 3% to 35% (13–17). Re-surgery is associated with higher morbidity (18–20). The proponents of prophylactic neck dissection suggest that it not only decreases recurrence, hence avoiding the morbidity associated with re-surgery, but also helps in accurate staging. However, others propose that performing unnecessary neck dissection is associated with morbidity in the form of recurrent laryngeal nerve paralysis and hypoparathyroidism. Morbidity in this cancer with an excellent outcome is not acceptable. Therefore, it is essential to assess the nodes correctly. Clinical examination has low sensitivity as metastatic nodes can be sub-centimetric, and areas such as the central compartment are not amenable to meticulous palpation. Therefore, imaging plays a pivotal role in the accurate assessment of the neck nodes of patients.

**Table 1:** Meta-analyses comparing outcomes of central compartment clearance.

Meta-analysis	Survival outcomes	Morbidity	Conclusion
Chisholm et al. (2009) (5)	—	Higher rate of temporary hypocalcemia	No increase in permanent morbidity
Zetoune et al. (2010) (6)	CND does not reduce the recurrence	—	No benefit over TT
Shan et al. (2012) (7)	Identical locoregional recurrence rate	Temporary hypocalcemia with CND	TT results in less surgical morbidity
Lang et al. (2013) (8)	Reduced risk of LRR	Higher temporary hypocalcemia	LRR risk reduction could be due to high RAI ablation or selection bias
Wang et al. (2013) (9)	Trend toward lower recurrence	No difference in long-term complication	Safe in high-volume centers
Zhao et al. (2017) (10)	Significantly reduces locoregional recurrence in the central compartment but no significant difference in the lateral compartment	Significantly higher odds of temporary hypocalcemia, permanent hypocalcemia, and increased overall morbidity	Lesser locoregional recurrence but an increased rate of hypocalcemia
Hughes et al. (2018) (11)	Effect on locoregional recurrence rates uncertain	Permanent hypoparathyroidism is more common but no significant difference in RLN palsy rates	May change the postoperative adjuvant RAI treatment
Liu et al. (2019) (12)	Overall recurrence is significantly lower; lower for the central compartment but not for the lateral compartment	—	Reduction in the risk of recurrence Prevents central compartment recurrence

CND, prophylactic central neck dissection; TT, total thyroidectomy; LRR, locoregional recurrence; RAI, radioactive iodine.

The most accepted and recommended imaging modality worldwide is ultrasound (US). However, it has certain limitations, such that it is operator dependent, cannot be used to image mediastinal and retropharyngeal nodes, which may have a high incidence of metastasis in thyroid cancer. The diagnostic accuracy is poor in assessing even the central compartment due to the anatomical location and overlying thyroid gland. A recently published meta-analysis of 19 studies with over 4,000 patients showed that the US pooled sensitivity and area under the curve (AUC) in assessing central compartment nodes were 0.33 and 0.69, respectively, compared with those in the lateral compartment, which were 0.70 and 0.88, respectively (21).

Attempts have been made to assess the accuracy of various cross-sectional imaging (22–24). However, the limitations of these modalities include low sensitivity, high cost, and precarious availability. A recently published meta-analysis of 38 articles with 6,285 patients compared 12 modalities, namely, US, computed tomography (CT), US + CT, contrast-enhanced US (CEUS), magnetic resonance diffusion tensor imaging (MRI), F-18 fluorodeoxyglucose positron emission tomography/CT (PET/CT), 131I whole-body (IWBC), US + CEU, strain elastography (SE-US), fine-needle aspiration thyroglobulin (FNA-Tg), US-guided fine-needle aspiration cytology (FNAC), and FNAC–FNA-Tg. The results showed that the sensitivity was highest for US + CT, and the specificity was highest for FNAC (25). Research has been directed to evaluate the role of contrast-enhanced CT (CECT) in assessing neck nodes (26–28). The advantages of CECT are its accessibility and availability of acquired images for interpretation. Moreover, it provides detailed anatomical location of the disease with respect to the surrounding landmark structures. However, it has limited accuracy in evaluating small thyroid nodules. The current American Thyroid Association guidelines recommend performing CECT in the presence of clinically bulky neck nodes and locally advanced thyroid cancers (3). The role in the routine assessment of all differentiated thyroid cancers remains unclear. We conducted a study at our center to evaluate the incremental value of CT to the US in detecting nodal metastasis in WDTC.

## Materials and methods

The institutional ethics committee approved this study, and the electronic medical records of 926 patients who underwent surgery at our center between 1 January 2013 and 30 November 2015 were retrieved. The final analysis was performed on 43 patients with 314 lymph nodal levels, who had both CT and US prior to surgery and whose level-wise lymph node histology was available. Two radiologists AM and NS reviewed the images independently while being blinded to the histopathological findings. The majority of the surgeries were performed by a single surgeon (AKD) who was assisted by a lead author (RV), and level-wise sampling of the nodes was performed in a standardized manner. The level-wise nodes were sent in separate packets for histopathological examination. The surgical and radiological findings were recorded and corroborated by a single investigator (RV) in a pre-specified proforma to maintain uniformity. Patient demographics were extracted from the electronic medical records, such as age, gender, clinical examination, FNAC, previous treatment, and surgical extent.

### Ultrasonography

US was performed on GE Voluson E8 with linear transducer at 7–18 MHz. Both the lateral and central compartments defined according to the AJCC were assessed. The central compartment (level VI) was scanned from the submental area to the sternal notch. The lateral compartment (levels II–IV) was scanned from the mandible to the clavicle and the posterior compartment (level V) along the posterior border of the sternocleidomastoid to the anterior border of the trapezius muscle. The following features were documented to differentiate between the metastatic and benign node: loss of fatty hilum, hypoechogenicity, cystic or necrotic changes, calcification, and size >5 mm in short-axis diameter with a rounded shape. The radiologist interpreting the US findings was blinded to the CT findings.

### Computed tomography

All patients underwent CT scans using the 16-slice multidetector CT scanner (Somatom Sensation 16, Siemens Healthcare). Unenhanced and enhanced scans were obtained for reconstruction slice thickness of 3 mm in axial and coronal images. The slices were acquired at 3 mm thickness and reconstructed at 0.75 mm thickness. The contrast-enhanced scans were obtained after injecting 50–90 ml of an iodinated non-ionic contrast agent at 1.8 ml/s using an automated injector with a scan delay time of 45 s. The scans were assessed on special BARCO Monitors (Barco Electronic Systems Pvt. Ltd.), wherein multiplanar reformations were obtained (to view images in the sagittal and coronal planes). The contrast-enhanced scans were obtained 45 s after intravenous contrast injection, which was the late arterial/early venous phase. The following criteria were documented to detect malignancy: strong enhancement without hilar vessel enhancement, heterogeneous enhancement, calcification, and cystic or necrotic changes. The radiologist analyzing the CT scan was blinded to the US findings.

**Figure 1:**
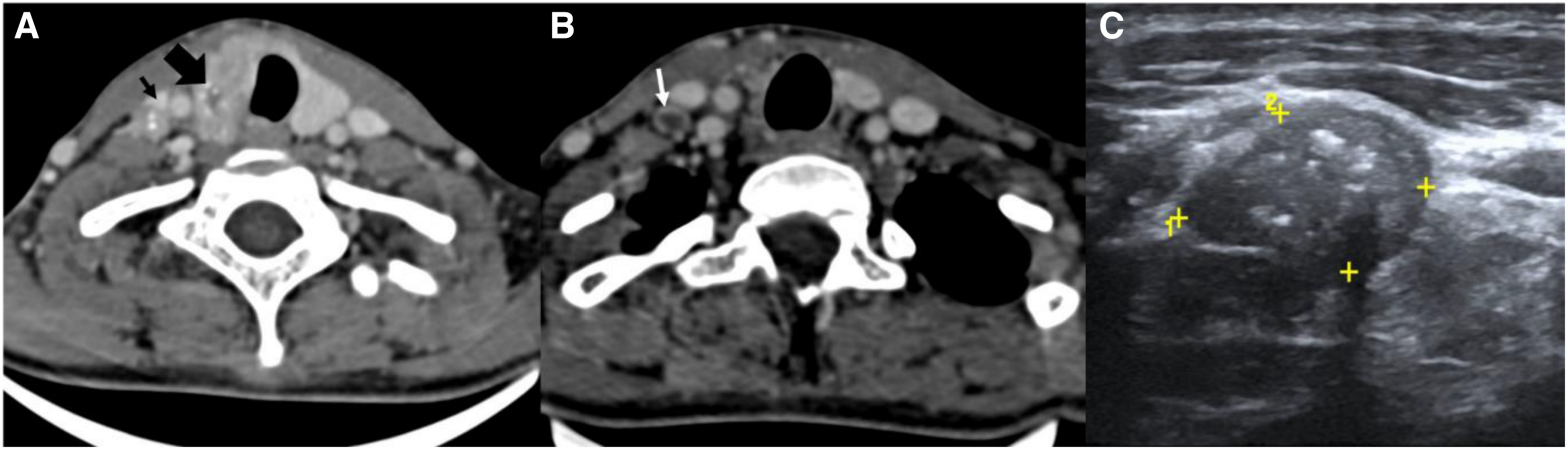
CT scan (**A**, **B**) and USG images (**C**) of the neck of a known case of papillary Ca thyroid. (**A**) CT scan images showing a heterogeneously enhancing thyroid mass (thick black arrow) with microcalcifications within and a similar morphology neck node at the right level IV (thin black arrow). (**B**) CT scan image of the same patient at a lower level showing a peripherally enhancing node with central necrosis (white arrow). (**C**) USG image of the same patient showing a metastatic node at the right level IV with microcalcifications within.

The image interpretation is shown in Figure 1.

• Benign: the absence of any suspicious feature.

• Indeterminate: the presence of any suspicious feature.

• Metastatic: the presence of two or more suspicious features.

The scans were reviewed by two radiologists independently, namely, AM and NS, and were reviewed and discussed together if there was a discrepancy in opinion.

### Surgery

Surgery was performed according to the standard protocol. Central compartment clearance was performed from the hyoid to the innominate artery and laterally up to the carotid sheaths. For selective neck dissection, levels II, III, and IV were sampled and sent for a frozen section. If metastasis was reported at levels II-IV on frozen section then level V clearance was also done. In addition, the patients with nodal metastasis confirmed by FNAC underwent level II–V clearance up front. The level-wise nodes were sent in separate packets for histopathology evaluation.

### Histopathology

For the histopathological examination, the nodes less than 1 cm were embedded in whole, and a single section was studied; the nodes measuring 1–2.5 cm were bisected along the long axis with both halves submitted for examination, those larger than 2.5 cm were sectioned serially, and a minimum of two sections were studied.

### Statistical considerations

The data were entered and analyzed using SPSS version 20. The sensitivity, specificity, positive predictive value (PPV), negative predictive value (NPV), and accuracy of US alone, CT alone, and both in predicting level-wise nodal metastasis were calculated for the overall, lateral and central compartments. The histopathology report was taken as the gold standard for comparison. The McNemar test was performed to compare the sensitivity and specificity; a *p*-value < 0.05 was considered significant.

## Results

### Demography

A total of 43 patients were analyzed in this study, of whom 27 were females and 16 were males. The mean age was 40.79 (12–79) years. FNAC was performed on 41 patients. It was suggestive of papillary carcinoma (TBS VI) in 32 patients and suspicious of papillary (TBS V) carcinoma in nine patients. A slide review of previous surgery in the remaining two patients showed papillary carcinoma. In total, 17 patients were clinically node-positive in the lateral neck. Central compartment nodes were not palpable in any patient.

### Surgery

A total of 40 patients underwent surgery for thyroid primary, and three patients underwent only neck dissection. For thyroid primary, 35 total and five completion thyroidectomies were performed. In addition, 39 patients underwent central compartment clearance, of whom 31 underwent bilateral neck dissection, four underwent right-sided neck dissection, and four underwent left-sided neck dissection. Furthermore, 43 patients underwent lateral neck dissection, of whom 31 were bilateral, four were right selective neck dissections, and eight were left selective neck dissections. Preoperative cords were bilaterally mobile in 36 patients, six patients had right-side impairment or palsy, and one patient had left-side impairment or palsy.

### Imaging and histopathology

A total of 516 nodal levels were analyzed in the US and CT. Histopathology was available for 314 nodal levels. Of the 314 nodes, 117 showed metastasis, and 197 were benign. US impression was malignant in 85 levels and benign in 229 levels. CT impression was malignant in 158 levels and benign in 156 levels. US + CT impression was malignant in 166 levels and benign in 148 levels. US, CT, and US + CT correctly diagnosed 63, 95, and 99 metastatic nodal levels.

### Histopathology

A total of 314 nodal levels were reported on histopathology. Overall, regional metastasis was present in 31/43 (72.1%) patients. In the lateral and central compartments, 25/43 (58.1%) and 24/39 (61.5%) patients had metastatic nodes, respectively.

### Overall

The sensitivity of CT was significantly higher than that in US, i.e., 81.20% and 53.85%, respectively (*p* < 0.001). The sensitivity of US + CT was significantly higher than that in US alone, i.e., 84.62% and 53.85%, respectively (*p* < 0.001). However, the difference between the sensitivity of CT and US + CT was not statistically significant (*p* = 0.125).

The specificity of US was significantly higher than that of CT, i.e., 88.83% and 68.02%, respectively (*p* < 0.001). The specificity of US was higher compared with that of US + CT, 88.83% and 65.99%, respectively (*p* < 0.001). The difference between the specificity of CT and US + CT was not statistically significant (*p* = 0.125) (Table 2).

**Table 2:** Sensitivity, specificity, NPV, and PPV of US, CT, and US + CT in the overall assessment of lymph node metastasis.

	Sensitivity (%)	Specificity (%)	PPV (%)	NPV (%)
US	53.85	88.83	74.12	76.42
CT	81.20	68.02	60.13	85.90
US + CT	84.62	65.99	59.64	87.84

The accuracy of the US was 75.80%, the CT scan was 72.93%, and the US + CT scan was 72.93%.

### Lateral compartment

The sensitivity of CT was significantly higher than that of US, 80.72% and 56.63%, respectively (*p* < 0.001). The sensitivity of US + CT was significantly higher than that of US alone, 84.34% and 56.63%, respectively (*p* < 0.001). However, the difference between the sensitivity of CT and US + CT was not statistically significant (*p* = 0.25).

The specificity of the US was significantly higher than that of CT, 91.41% and 70.55%, respectively (*p* < 0.001). The specificity of US was higher compared with that of US + CT, 91.41% and 68.71%, respectively (*p* < 0.001). The difference between the specificity of CT and US + CT was not statistically significant (*p* = 0.25) (Table 3).

**Table 3:** Sensitivity, specificity, NPV, and PPV of US, CT, and US + CT in the assessment of lateral compartment lymph node metastasis.

	Sensitivity (%)	Specificity (%)	PPV (%)	NPV (%)
US	56.63	91.41	77.05	80.54
CT	80.72	70.55	58.26	87.79
US + CT	84.34	68.71	57.85	89.60

The accuracy of the US was 79.67%, the CT scan was 73.98%, and the US + CT scan was 73.98%.

### Central compartment

The sensitivity of CT was significantly higher than that of US, 82.35% and 47.06%, respectively (*p* < 0.001). The sensitivity of US + CT was significantly higher than that of US alone, 85.29% and 47.06%, respectively (*p* < 0.001). However, the difference between the sensitivity of CT and US + CT was not statistically significant (*p* = 1).

The specificity of the US was significantly higher than that of CT, 76.47% and 55.88%, respectively (*p* = 0.039). The specificity of US was higher compared with that of US + CT, 76.47% and 52.94%, respectively (*p* < 0.01). The difference between the specificity of CT and US + CT was not statistically significant (*p* = 1) (Table 4). The accuracy of the US was 61.76%, the CT scan was 69.12%, and the US + CT scan was 69.12%.

**Table 4:** Sensitivity, specificity, NPV, and PPV of US, CT, and US + CT scan in the assessment of central compartment lymph node metastasis.

	Sensitivity (%)	Specificity (%)	PPV (%)	NPV (%)
US	47.06	76.47	66.67	59.09
CT	82.35	55.88	65.12	76.00
US + CT	85.29	52.94	64.44	78.26

The specificity of US, CT, and US + CT was higher for the lateral compartment than for the central compartment.

### Follow-up and disease outcomes

At the median follow-up of 59 (0.03–106) months, eight patients had disease recurrence. Two patients had nodal recurrence, one patient had locoregional recurrence, one patient had nodal recurrence with skin nodule, and four patients had distant metastasis. At the last follow-up, 36 patients were alive and disease-free, six patients were alive with disease, and one patient was dead due to an unknown cause.

## Discussion

The incidence of regional metastasis in papillary thyroid cancer has been reported as 30%–84% (29–32). The first echelon of drainage is the central compartment. The incidence of occult metastasis in the central compartment ranges from 37% to 41% (33, 34). The incidence of metastasis, even in microcarcinoma, has been reported to be 64.1% in the central compartment and 44.5% in the lateral compartment (35). The overall incidence of nodal metastasis in our study was 72.1%. The incidence of the central and lateral compartments was 61.5% and 58.1%, respectively.

US is the initial modality for the assessment of regional metastasis. The sensitivity of the US reported in the literature is approximately 46%–92%. The sensitivity for the central compartment is lower than that for the lateral compartment. The specificity ranges between 73% and 100% (36–40). The probability of missing the disease is high in the US due to low sensitivity. In our study, the overall sensitivity, specificity, PPV, and NPV of the US were 53.85%, 88.83%, 74.12%, and 76.42%, respectively. The sensitivity, specificity, PPV, and NPV were 56.63%, 91.41%, 77.05%, and 80.54% for the lateral compartment and 47.06%, 76.47%, 66.67%, and 59.09% for the central compartment, respectively (Tables 2–4).

There are various reasons for the low sensitivity of the US. First, US features considered have varied accuracy in predicting the nature of the node (41, 42). Size as an isolated criterion has lower accuracy compared with microcalcification and necrosis. Unlike squamous cell carcinoma, even a sub-centimeter node can harbor metastasis in the case of papillary thyroid cancer (32). Second, the US is observer-dependent, and studies have shown that there exists a significant inter-observer variation (43). More experienced sonologists or surgeons have higher accuracy in detecting nodal disease than someone working in low-volume centers with limited experience. Third, the US has a technical limitation in assessing the central compartment nodes (44). The presence of overlying thyroid, clavicle, and sternocleidomastoid decreases the sensitivity of the US in detecting central compartment metastasis. In addition, certain areas such as the parapharyngeal and mediastinal nodes are not accessible for US examination. Although we have not compared US with CT scan for mediastinal and parapharyngeal nodes in our study, the US has low sensitivity by design due to penetration limitations and shadowing from the clavicle, lung air spaces, and thyroid gland.

The CT scan has the advantage of being objective and images available for interpretation. It overcomes the limitation of the US in assessing the central compartment. It is also helpful in imaging nodes in the upper thorax, which are difficult to examine in the US and are routinely missed. Studies have compared the sensitivity and specificity of the US with CT scans. The sensitivity in most studies is reported to be higher for CT scans. This difference is specially marked for the central compartment. However, the results on specificity are inconsistent (27, 45–54). High sensitivity makes CT an excellent screening tool for detecting regional metastasis. The overall sensitivity, specificity, PPV, and NPV of the CT scan were 81.20%, 68.02%, 60.13%, and 85.90%, respectively. The sensitivity, specificity, PPV, and NPV were 80.72%, 70.55%, 58.26%, and 87.79% for the lateral compartment and 82.35%, 55.88%, 65.12%, and 76.00% for the central compartment, respectively (Tables 2–4). The limitation of CT scans is the low specificity. High false-positive results may over-stage the disease and result in unwarranted neck dissection.

The issue with a CT scan that constrains its use in thyroid carcinoma assessment is the administration of iodinated contrast. It is often presumed that it may affect the efficacy of radioiodine ablation in the postoperative period by hindering its entrapment by the thyroid. It has been shown that urine iodine excretion 1 month after the preoperative CT scan is the same as that 6 months after the scan, challenging the concept of delaying the radioactive iodine (RAI) for 3–4 months after the scan (45, 55). Second, the role of RAI is limited in low and intermediate-risk patients. It can be safely avoided in patients with small primaries < 1 cm and micrometastasis in nodes <5 in number without other high-risk features. Therefore, a CT scan can be done in most patients without affecting the radioactive iodine ablation efficacy as it is either not warranted or has no proven survival benefit. A CT scan can, therefore, be used for imaging these cancers. An established role is in high-risk patients with extrathyroidal extension, >4 cm in size, and large nodal metastasis. In locally advanced cancer, a CT scan is the modality of choice for disease assessment, precluding this argument in these cases (2, 3).

The highest sensitivity in the literature is reported with the US + CT combination (Table 5) (27, 45, 47, 48, 50, 52, 56). Therefore, the combination is best to assess both the lateral and central neck for metastasis. In our study, the sensitivity was highest for combined US + CT for the overall, lateral and central compartments, 84.62%, 84.34%, and 85.29%, respectively. A recently published meta-analysis of 11 studies with 6,261 thyroid cancer patients reported the diagnostic accuracy of US + CT in assessing neck node metastasis. The sensitivity and specificity to detect central compartment metastasis were 0.57 and 0.82, respectively; 0.89 and 0.79, respectively, for the lateral compartment; and 0.73 and 0.80, respectively, for all compartments (57).

**Table 5:** Studies comparing the sensitivity and specificity of US, CT, and US + CT in assessing overall, central and lateral compartments in thyroid cancers.

	Number (*n*)	Overall		Central compartment		Lateral compartment	
		Sensitivity	Specificity	Sensitivity	Specificity	Sensitivity	Specificity
Kim et al. (2008) (27)	165 patients	US + CT	Similar for US and CT higher than US + CT	US + CT	Similar for US and CT higher than US + CT	US + CT	US + CT similar to US and lower than CT
Lesnik et al. (2014) (45)	162 patients	—	—	CT better than US, highest for US + CT	US and CT comparable and higher than US + CT	US and CT similar and highest for US + CT	US and CT comparable and higher than US + CT
Yoon et al. (2011) (46)	122 lateral cervical lymph nodes of 113 patients	—	—	—	—	US + CT	US
Lee et al. (2013) (47)	252 patients	US + CT	US	US + CT	US	US + CT	US
Choi et al. (2009) (48)	299 patients	—	—	highest for US + CT	Similar for US and CT higher than US + CT	Highest US + CT Comparable to US	CT
Ahn et al. (2008) (49)	37 patients	CT	US	CT	US	CT	US
Kim et al. 2017 (50)	6,577 central neck levels 3,668 PTC patients	—	—	CT + US	US	—	—
Choi et al. (2010) (51)	589 patients	CT	CT marginally higher than	US	CT marginally higher	CT	US marginally higher
Suh et al. (2017) (52)	1,691 patients	US + CT	US	US + CT similar to CT	US	US + CT	CT
Xing et al. (2020) (53)	5,656 patients	CT higher than US	US higher than CT	CT higher than US	US higher than CT	CT higher than US	US higher than CT
Yang et al. (2022) (56)	11,601 lymph nodes	US + CT	US	US + CT	US	US + CT	US
Alabousi et al. (2022) (54)	12,771 lymph nodes	US and CT similar	US and CT similar	CT higher than US	US higher than CT	US and CT similar	US and CT similar
Albuck et al. (2023) (58)	15,014 lymph nodes	CT + US better	US better	CT and CT + US similar and better than US	CT and US similar and slightly higher than CT + US	CT + US better than CT or US alone	CT greater than US and CT + US

Our center is a tertiary cancer center, and we perform more than 300–350 thyroid surgeries in a year. The strength of this study is that the image analysis is done by a group of radiologists with adequate experience in thyroid imaging at a high-volume center. Most surgeries were performed by a single senior surgeon and assisted by the candidate in a standardized manner after studying the images with radiologists. The study provides a level-wise assessment of the two imaging modalities and their combination, which has been compared with the histopathology in all patients. In addition to the correlation with the histopathology, the study also provides a long-term follow-up of the patients who have undergone surgery, which is not provided in most other similar studies. A small number of patients is the limitation of the study as a CT scan is not performed in all the patients who undergo surgery for early disease.

Although the high sensitivity of CT scans makes it an ideal imaging for screening, the low specificity is the obvious limitation. US with high specificity and low sensitivity is likely to miss metastatic nodes, especially in the central compartment. Therefore, neither of the imaging modalities alone is good enough to assess the neck.

Therefore, we conclude that the sensitivity of CT and US + CT was significantly higher than the US in detecting the overall, lateral compartment and central compartment regional metastasis. The specificity of US was significantly higher compared with CT and US + CT in detecting the overall, lateral compartment and central compartment regional metastasis. The role of US and CT scans is complementary in assessing neck node metastasis. However, a large numbered prospective study is needed to accurately determine the role of these imaging modalities in WDTC.

## Data Availability

The raw data supporting the conclusions of this article will be made available by the authors, without undue reservation.
